# Enteric Nervous System Alterations in Inflammatory Bowel Disease: Perspectives and Implications

**DOI:** 10.3390/gidisord6020025

**Published:** 2024-03-28

**Authors:** Shubhankar Suman

**Affiliations:** Department of Oncology, Lombardi Comprehensive Cancer Center, Georgetown University Medical Center, Washington, DC 20057, USA;

**Keywords:** enteric nervous system, inflammatory bowel disease, gut–brain axis

## Abstract

The enteric nervous system (ENS), consisting of neurons and glial cells, is situated along the gastrointestinal (GI) tract’s wall and plays a crucial role in coordinating digestive processes. Recent research suggests that the optimal functioning of the GI system relies on intricate connections between the ENS, the intestinal epithelium, the immune system, the intestinal microbiome, and the central nervous system (CNS). Inflammatory bowel disease (IBD) encompasses a group of chronic inflammatory disorders, such as Crohn’s disease (CD) and ulcerative colitis (UC), characterized by recurring inflammation and damage to the GI tract. This review explores emerging research in the dynamic field of IBD and sheds light on the potential role of ENS alterations in both the etiology and management of IBD. Specifically, we delve into IBD-induced enteric glial cell (EGC) activation and its implications for persistent enteric gliosis, elucidating how this activation disrupts GI function through alterations in the gut–brain axis (GBA). Additionally, we examine IBD-associated ENS alterations, focusing on EGC senescence and the acquisition of the senescence-associated secretory phenotype (SASP). We highlight the pivotal role of these changes in persistent GI inflammation and the recurrence of IBD. Finally, we discuss potential therapeutic interventions involving senotherapeutic agents, providing insights into potential avenues for managing IBD by targeting ENS-related mechanisms. This approach might represent a potential alternative to managing IBD and advance treatment of this multifaceted disease.

## 1. Introduction

Inflammatory bowel disease (IBD) is a group of chronic inflammatory conditions of the gastrointestinal (GI) tract characterized by periods of inflammation and remission [1,2]. Typical symptoms of IBD include fatigue, weight loss, bloody diarrhea, tenesmus, abdominal pain/cramps, and urgency to have bowel movements. The two primary subtypes of IBD are Crohn’s disease (CD) and ulcerative colitis (UC) [1]. CD can affect any part of the digestive tract and often displays transmural inflammation, whereas UC primarily affects the colon and rectum and is characterized by continuous mucosal inflammation [3]. As our understanding of IBD continues to expand, it is now widely recognized that IBD has not only local but also systemic effects [4,5]. Particularly, recent reports have implicated the role of an altered gut–brain axis (GBA) and higher incidence of mental disorders in patients with IBD [6,7]. The GBA involves two-way communication between the central nervous system (CNS) and the enteric nervous system (ENS), connecting the emotional and cognitive areas of the brain to the functions of the GI tract [8]. A functional GBA is required for regulation of digestion, nutrient absorption, and normal functioning of the gut; the dysregulation of this axis in IBD adds another layer of complexity in the pathophysiology of this disease that needs to be addressed to reduce the incidence of mental disorders in these patients [9,10]. Moreover, IBD-mediated changes in the ENS could alter bi-directional communication through the GBA [11], potentially exacerbating gut symptoms in individuals with IBD. Recognizing the impact of IBD-mediated changes in the ENS on GBA communication underscores the importance of addressing this axis in the clinical management of IBD, offering potential avenues for more effective therapeutic interventions and improved outcomes for affected individuals.

## 2. Central Role of ENS in Gut–Brain Axis

**Figure 1.**
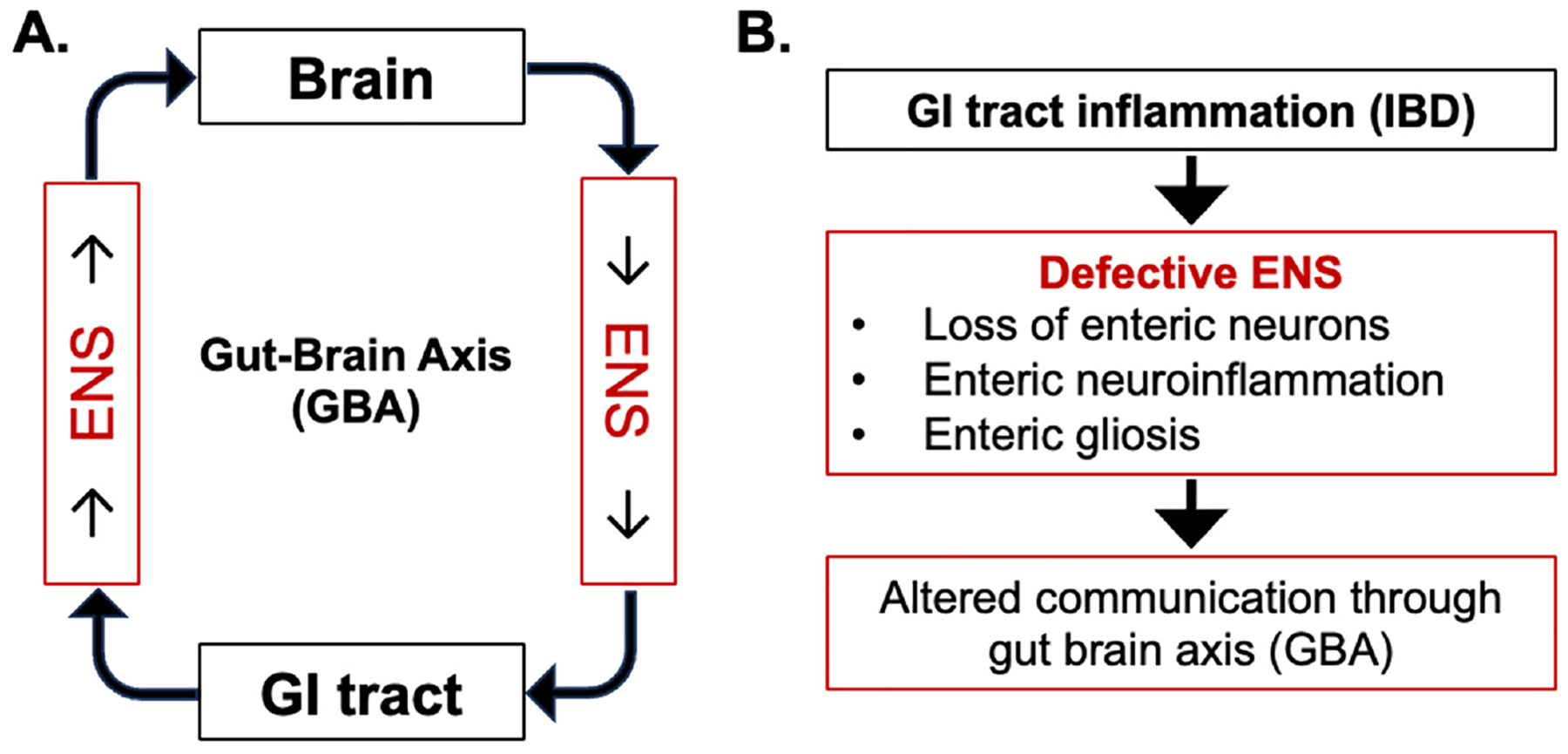
Central role of enteric nervous system (ENS) in communication through Gut–Brain Axis (GBA). (**A**) Normal bi-directional communication between the GI tract and brain. (**B**) Alterations in bi-directional communication due to GI inflammation-induced enteric neuronal loss, neuroinflammation, and gliosis during inflammatory bowel disease (IBD).

The ENS is composed of millions of neurons and a vast network of interconnected ganglia that play a crucial role in regulating processes, such as gut secretion, motility, and blood flow [12]. Sensory neurons in the ENS detect various stimuli and relay this information to the intrinsic neurons, which can then initiate appropriate responses [13]. These neurons are organized into two main plexuses. i.e., the myenteric plexus (located between the longitudinal and circular muscle layers of the GI tract) and the submucosal (Meissner’s) plexus (found in the submucosa of the gut wall) [14,15]. Additionally, interneurons in the ENS process and integrate sensory information and help coordinate the responses of the ENS. While ENS can regulate various gut functions independently of the CNS, the GBA is implicated in the bi-directional relationship between the gut and mental health that influences various aspects of our physical and emotional well-being [16,17]. Changes in the gut environment can influence behavior, and conversely, stress and emotions can impact gut function (Figure 1A). The ENS is a key player in this complex interplay [8]. Several mechanisms of the bi-directional communication between the ENS and the brain are known, including (1) communication between the ENS and the CNS through sensory and motor neurons, where sensory neurons sense changes in the gut environment and then communicate to the CNS via motor neurons, providing a constant stream of information about the state (such as pH and nutrient levels) of the digestive system. Specifically, motor neurons in the ENS gather inputs from diverse origins, encompassing sensory neurons within the ENS and signals from the CNS conveyed through sympathetic and parasympathetic nerves. This complex interconnection facilitates synchronized regulation of GI activities, comprising peristalsis, secretion, and blood circulation; (2) bi-directional communication between the ENS and the CNS through the vagus nerve, i.e., a part of the autonomic nervous system (ANS). Notably, sensory neurons in the GI detect changes in the environment (i.e., presence of nutrients) and transmit these signals via the vagus nerve to the brainstem, allowing the CNS to modulate digestive functions based on sensory input. Conversely, the CNS can influence ENS activity by sending signals associated with emotional states like stress or anxiety through the vagus nerve, sometimes leading to symptoms like nausea or changes in bowel habits; (3) communication through neurotransmitters (chemical messengers) such as serotonin, dopamine, and acetylcholine, which play important roles in regulating gut function; (4) both the ENS and the CNS produce, respond to, and regulate the release of hormones involved in digestion and metabolism; and (5) interaction with gut microbiota and the immune system in the gut through microbial metabolites and signaling molecules that affect the ENS and brain function. Notably, the gut microbiome is known to produce a variety of signaling molecules, including neurotransmitters (like serotonin and dopamine) and neuromodulators (like gamma-aminobutyric acid or GABA). These molecules can interact with the CNS, affecting gut motility, intestinal permeability, and even the central nervous system [18–22]. These functions highlight the intricate role of the ENS not only in GI health but also in emotional well-being and cognitive function.

## 3. Enteric Nervous System Alterations in IBD

Chronic inflammation, damage to the gut mucosa, and immune system dysregulation in IBD can lead to structural and functional alterations within the ENS, therefore potentially altering communication through the GBA (Figure 1B) [23–25]. Myenteric plexitis has been reported in a majority of CD (75%) and UC (56%) cases [26]. Patients with CD often display defects in enteric glial networks [27,28], often associated with neuronal damage, loss of neural connections, and neuroinflammation [29]. Routine pathological assessments often describe: (i) damage of nerve fibers; (ii) hypertrophy of neuronal cell bodies; and (iii) enteric glial cell (EGC) hyperplasia [30]. Therefore, ENS alterations in patients with IBD can generally be described as enteric neuronal cell effects as well as the responses of other types present in the ENS, including ECGs.

### 3.1. Pathophysiology of IBD and Alterations to Enteric Neurons

Over time, IBD can escalate to enteric neuropathy, further disrupting GI functions and leading to a range of digestive issues [31–33]. Alterations in enteric neurons are evident in both CD and UC, but they are more pronounced in patients with CD, particularly within the mucosa, submucosa, and myenteric plexus of the ileum and colon. Patients with CD often experience myenteric and submucosal plexitis [23,34]. Despite these distinctions in clinical aspects of IBD, enteric neurons are significantly affected during GI inflammation, involving changes in the structure of the enteric neuronal network as well as in its neurotransmitter signaling. Patients with IBD exhibit abnormalities in the size and number of nerve bundles and ganglia, leading to dysmotility, altered secretion, and heightened sensation of pain and discomfort [23]. Moreover, a study using mouse models of colitis has demonstrated that enteric neuronal density could affect the severity of intestinal inflammation [35]. Additionally, enteric neurons also display changes in morphology and function associated with neuronal plasticity, synaptic connectivity, and neuronal excitability [20,36]. While some of these changes may be adaptive, others can exacerbate GI dysfunction, contributing to abdominal pain and discomfort [37–40].

At the molecular level, using single-cell RNAseq analysis, it has been reported that the human ENS expresses risk genes for neuropathic and inflammatory diseases. Moreover, many IBD risk genes, including *BTBD8* (BTB Domain Containing 8), *GRP* (Gastrin-Releasing Peptide), *JAZF1* (Juxtaposed with Another Zinc Finger Protein 1), *NDFIP1* (Nedd4 Family-Interacting Protein 1), *PLA2R1* (Phospholipase A2 Receptor 1), and *CNTNAP2* (Contactin-Associated Protein 2), were found to be enriched in the ENS, which suggests a potential role of the ENS in IBD pathogenesis [41]. GI inflammation during IBD can also cause neuroinflammation in enteric neurons [42–44] and degeneration in enteric ganglia [45,46]. In concurrence, variations in the cell number and neuronal profile of inflamed areas compared to healthy tissue have also been reported earlier [47].

The mechanism of neuronal cell death is not fully understood but appears to involve interactions with the mucosal immune system. Therapeutic interventions targeting leukocytes have shown efficacy in murine models and in human patients by reducing inflammation and improving gut function. Additionally, resolution of inflammation is associated with gradual improvement in gut motility [48,49]. Further, in response to neuronal inflammation, immune cells infiltrate the gut wall and produce pro-inflammatory cytokines and other factors that can exacerbate neuronal dysfunction and damage. Aberrant neuroimmune interactions in the GI tract may also contribute to the perpetuation of inflammation and tissue damage [50].

Enteric neurons can release neurotransmitters and neuropeptides that modulate immune cell function, and conversely, immune cells can produce factors that affect neuronal activity [20,23]. In the rodent gut, inflammation significantly affects cholinergic neurons, which are crucial for excitatory functions in enteric neurons, leading to reduced acetylcholine (Ach) release. This decrease may stem from alterations in the expression of synaptic vesicle proteins necessary for neurotransmitter release, such as the selective reduction of neuronal calcium sensor 1 during trinitrobenzene sulfonic acid (TNBS)-induced colitis. Additionally, serotonin, a key gastrointestinal hormone and neurotransmitter involved in initiating peristaltic activity, is now recognized as a pro-inflammatory neurotransmitter [23,51–53], which is elevated in patients with CD. In view of existing evidence demonstrating both structural and functional alterations in enteric neurons during IBD, a detailed understanding of the pathophysiological role of enteric neurons in IBD is important for developing novel therapeutic strategies to alleviate symptoms and improve disease outcomes.

### 3.2. Pathophysiology of IBD and Alterations to Enteric Glial Cells (EGCs)

EGCs provide structural and metabolic support for enteric neurons through a close association with neuronal cells and also express receptors for neurotransmitters such as serotonin, gamma-aminobutyric acid (GABA), and glutamate, allowing them to respond to neuronal activity and regulate synaptic transmission [37,54]. Additionally, EGCs also contribute to the maintenance of the intestinal epithelial barrier, which is compromised in IBD [55]. They regulate the expression of tight junction proteins and mucins, which are essential for epithelial integrity and protection against luminal pathogens [56]. EGCs can also interact with immune cells, such as mast cells and macrophages, and modulate their function through the release of cytokines and other signaling molecules [57,58]. IBD can disrupt this supportive role of EGCs, through activation of EGCs triggered by an increase in the levels of pro-inflammatory cytokines, microbial products, and overall tissue inflammation/damage. Involvement of EGCs’ activation in chronic and acute gut inflammation has been suggested earlier [59–62]. Both in vivo and in vitro studies have shown that EGCs can turn into reactive glia after encounters with IBD-associated stimuli such as bacterial lipopolysaccharide (LPS) and immune cell-derived pro-inflammatory factors including interleukin (IL)-1β, tumor necrosis factor alpha (TNFα), and interferon-gamma (IFN-γ) [63–66]. These cytokines can directly influence the function of enteric neurons, leading to alterations in neurotransmitter release, synaptic plasticity, and neuronal excitability [63,64]. Furthermore, EGCs have been implicated to have a harmful impact on CD-associated inflammation [67,68]. Particularly, EGCs are known to function as antigen-presenting cells (APCs) during IBD [69]. Another potential role of EGCs in GI inflammation could involve expression of T-lymphocyte costimulatory molecules [62,70]. Additionally, persistent activation of EGCs during GI tract inflammation is known to develop into gliosis that involves changes in glial cell structure and function, including hypertrophy (increased cell size) and increased expression of gliosis markers. Gliosis in the enteric plexus could alter both the ANS as well as GBA communication involving the ENS [59,71] which can adversely impact physiological processes such as gut motility, barrier function, and immune and hormonal responses known to contribute to flare-ups and the recurrence of IBD [65]. Additionally, activated EGCs are also known to cause enteric neuronal death during the acute phase of IBD [72]. These findings underscore the complex interplay between GI inflammation and ENS dysfunction in the pathogenesis of IBD. Therefore, understanding the influence of EGC activation on enteric neuronal function is critical in fully understanding the pathophysiology of IBD and identifying potential therapeutic targets.

## 4. EGC Senescence in the Etiology of IBD

Using animal models of IBD, increased BrdU (Bromodeoxyuridine) incorporation in EGCs has suggested the stimulation of cell proliferation in myenteric glia [73]. EGC proliferation in response to GI inflammation has also been demonstrated to accompany alterations in glial fibrillary acidic protein (GFAP) expression, morphological changes, the release of cytokines, and shifts in gene expression profiles [39,74–76]. Activated EGCs display gene and protein expression patterns similar to those in gliosis in the brain and can release pro-inflammatory cytokines, nitric oxide (NO), and reactive oxygen species (ROS) [60,77–79] and EGCs with higher levels of oxidative DNA damage as well as shortening of telomeres due to excessive proliferation can undergo cellular senescence (a state of irreversible cell cycle arrest) [80,81]. Further, IBD-induced loss of intestinal barrier function followed by bacterial infection can also induce senescence in EGCs [80,82]. Notably, pathogen-induced interferon-gamma (IFN-γ)-mediated responses orchestrated by EGCs have been shown to play a role in coordinating gut responses to pathogens. Furthermore, downstream cell lineages affected by the IFN-γ–EGC axis are implicated in IBD. The shared IFN-γ response signature between EGCs from patients with UC and helminth-infected mouse EGCs, coupled with associations between elevated Cxcl10 (C-X-C Motif Chemokine Ligand 10) levels and increased IBD risk, underscores the potential therapeutic significance of targeting enteric glia in the management of IBD [83].

**Figure 2.**
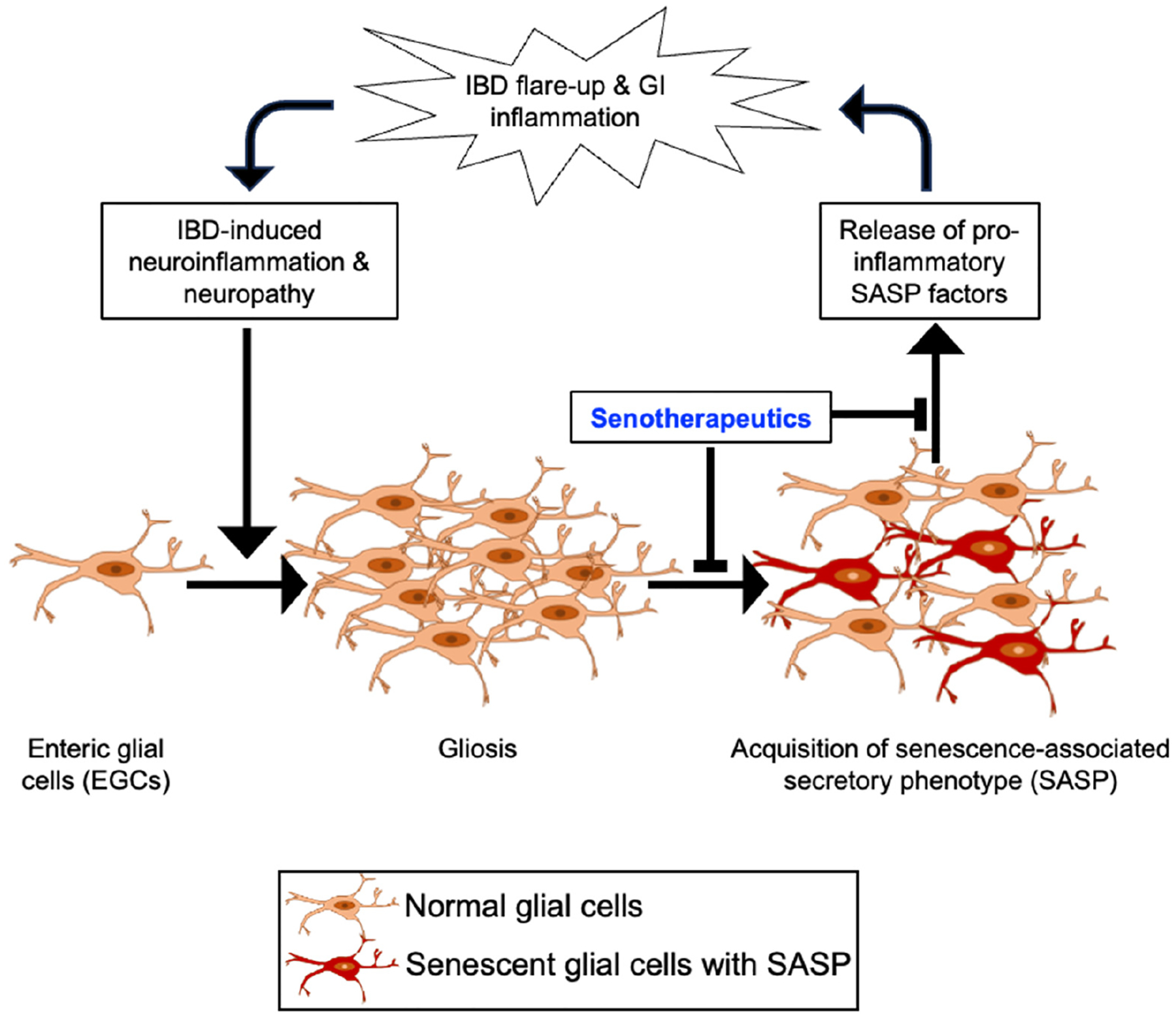
Schematic depiction of the potential role of enteric gliosis in inflammatory bowel disease (IBD) recurrence and the intervention approach using senotherapeutic agents. A vicious cycle from initial IBD to recurrent IBD could involve inflammation in the enteric nervous system (ENS), leading to persistent gliosis in the ENS via activation of enteric glial cells (EGCs). Activated glial cells undergoing senescence may secrete inflammatory mediators via acquisition of senescence-associated secretory phenotype (SASP), which can further exacerbate GI tract inflammation through the release of pro-inflammatory factors, contributing to the recurrence of IBD.

IBD manifestation often occurs at a relatively young age (<40 years). For CD and UC, the peak age of onset is typically between 15 and 30 years and 15 and 40 years, respectively. IBD recurrence (flare-ups) is largely dependent on lifestyle factors [84–87]. In contrast, late-onset IBD is initially diagnosed at the age of 60 or older and coincides with aging-associated systemic inflammation [87], where cellular senescence seems to be a central player in both IBD and systemic inflammation [88,89]. Cellular senescence is usually triggered after activation of the DNA damage response (DDR), and senescent cells often display increased expression of cell cycle checkpoint proteins (such as p16, p21, and p53) and exhibit resistance against cell death via activation of anti-apoptotic pathways [88,89]. Cellular senescence involves an irreversible cell replication arrest, resistance to apoptosis, and development of a pro-inflammatory secretory profile known as the senescence-associated secretory phenotype (SASP), which is known to contribute to inflammation, metabolic dysfunction, and stem cell impairments in the GI tract [88–91]. Evidence suggests that accumulation of senescent cells can have adverse effects on nearby cells as well as on the tissue microenvironment through acquisition of a senescence-associated secretory phenotype (SASP). While not all senescent cells exhibit a SASP, some do, releasing interleukins, cytokines, and chemokines that attract, activate, and anchor immune cells as well as pro-apoptotic factors, matrix metalloproteinases (MMPs), growth factors, reactive metabolites, bioactive lipids like saturated fatty acids and ceramides, bradykinins, prostanoids, micro-RNAs, and extracellular vesicles, among other factors [91,92]. Therefore, the buildup of senescent cells over a time span has the potential to induce SASP-mediated inflammation leading to tissue damage, suppression of the immune system, and secondary senescence in neighboring normal cells. Strategies aimed at delaying the accumulation or reducing the burden of senescent cells have been linked to prolonging and preventing inflammation-associated health conditions [90]. These advancements are now progressing towards clinical interventions and could offer transformative possibilities in the management of IBD. In the context of IBD and the ENS, senescent EGCs persisting in form of enteric gliosis are likely to cause chronic inflammation as well as inflammatory flare-ups by releasing their own set of inflammatory signals after acquiring SASP. Therefore, it is plausible that the pro-inflammatory and detrimental effects of glial SASP could contribute to chronic inflammation that may exacerbate IBD flare-ups after initial remission (Figure 2). Overall, the accumulation of SASP cells in the ENS has the potential to induce inflammation, tissue damage, and finally exacerbate IBD. Therefore, strategies targeting senescent cell accumulation could be promising in managing IBD.

## 5. Plausibility of Senolytics Intervention for the Management of IBD

Current clinical management of IBD typically involves a combination of therapies aimed at achieving and maintaining remission, alleviating symptoms, and preventing complications. Common therapeutic approaches involve use of aminosalicylate drugs such as sulfasalazine to treat mild to moderate IBD by reducing inflammation in the gut. Additionally, corticosteroids such as Prednisone and Budesonide are typically used to control inflammation during IBD flares. Further, immunomodulators like Azathioprine, 6-mercaptopurine, and methotrexate are also used to suppress the immune system’s response. For targeted therapy, monoclonal antibodies (such as, infliximab, adalimumab, vedolizumab, and ustekinumab) and Janus kinase (JAK) inhibitor (Upadacitinib) have also shown promising results for the treatment of moderate to severe IBD [93–98]. However, none of these drugs have been investigated for their efficacy on IBD-associated ENS complications but studies have rather focused on control of mucosal inflammation. Since the ENS is a central player in GBA communication, a detailed understanding of the IBD-associated alterations in the ENS could lead to development of targeted interventions offering novel therapeutic avenues for addressing both GI and mental health aspects of the IBD.

IBD is often characterized by periods of remission followed by disease recurrence and symptom flare-ups. Therefore, in the case of an IBD patient showing enteric gliosis, specific treatment options focused at targeting this issue might be helpful in reducing the risk of IBD flare-ups and IBD-associated chronic GI tract inflammation (Figure 2). Particularly, senescent and SASP EGCs can be specifically targeted using serotherapeutic approaches [99–101] including (a) senolytics, (b) senomorphics, and (c) SASP neutralizing antibodies (SNmAb). Senolytic agents can selectively induce death of senescent cells, whereas senomorphics are known to block the acquisition of SASP and production of inflammatory cytokines from senescent cells. Another alternative strategy is the use of a SASP neutralizing antibody (SNmAb) cocktail [99–102] that requires identification and neutralization of EGC-released SASP factor. Recently, a pro-inflammatory cytokine and a designated SASP factor, i.e., interleukin-1 (IL1), has been implicated in EGC activation and the onset of enteric gliosis, as well as in IBD [65,103]. Evidence exists to suggest the plausibility of these serotherapeutic-based interventions for the management of IBD, for example, (1) a senolytic combination of D + Q (Dasatinib and Quercetin) has been demonstrated to reduce intestinal inflammation [104]; (2) anti-diabetic and senomorphic drug metformin has been recently reported to protect against IBD [105]; and (3) a monoclonal antibody toward IL-1β (canakinumab) has also shown effectiveness among older patients with IBD [106]. Hoverer, future studies using senomorphics, senolytics, and SNmAb are required to establish the clinical efficacy and applicability in IBD patients with enteric gliosis.

## 6. Effects of Serotherapeutic Agents on Gut Dysbiosis

Gut dysbiosis is common among patients with IBD [107,108]. While gut dysbiosis is not the sole cause of IBD, it is believed to play a significant role in its development, progression, and recurrence [109,110]. Various cell types present within the GI tract function as a protective shield, keeping the host tissues separate from the luminal microbiome and also regulating inflammatory balance within the gut. GI tract inflammation can disrupt GI barrier functions, leading to heightened gut permeability and increased vulnerability to infections, with a concurrent rise in senescent cell load, including EGCs with SASP phenotype. Recent studies have also suggested a bi-directional association between the microbiome and senescent cell accumulation in the GI tract [111,112]. In view of EGCs’ role in coordinating gut responses to pathogens [83], it is imperative to assume that senotherapeutics will not only impact the accumulation of senescent/SASP cells in the ENS but can also address the adverse impact of gut dysbiosis during IBD. Interestingly, emerging data on senotherapeutics have also shown effects on the gut microbiome profile. Metformin can positively modulate the promotion and maintenance of a healthy microbiome in the gut as well as reduce the senescent/SASP cell load in the GI tract [113,114]. Moreover, Fisetin, a well-recognized senolytic drug, has also been shown to mitigate dextran sulfate sodium (DSS)-induced colitis by targeting senescence and inflammation with concurrent restoration of a healthy microbiome in the mouse GI tract [115]. Therefore, understanding the interplay between senescence, inflammation, and the microbiome opens avenues for novel therapeutic approaches in managing IBD, underscoring the significance of understanding and targeting these interconnected mechanisms for improved patient outcomes.

## 7. Conclusions

In conclusion, IBD-associated inflammation causes functional and structural changes in enteric neurons and ganglia that could potentially alter GBA communication. Additionally, persistent activation of EGCs can lead to gliosis, involving structural and functional changes in glial cells. This, in turn, may adversely impact physiological processes such as gut motility, barrier function, and immune and hormonal responses. Moreover, EGCs subjected to higher levels of oxidative DNA damage and telomere shortening due to excessive proliferation may undergo cellular senescence. The persistence of senescent EGCs and acquisition of SASP could play a pivotal role in perpetuating inflammation, potentially exacerbating IBD flare-ups even after the initial remission. Studying enteric gliosis-associated SASP (senescence-associated secretory phenotype) factors offers several potential benefits for characterizing novel IBD biomarkers and therapeutic targets. Identification of enteric gliosis-specific SASP factors in serum can identify novel molecules that can also be used as biomarkers for dysregulated GI health in patients with IBD. These biomarkers can also be used to assess response to therapy and guide adjustments in treatment regimens to achieve optimal outcomes for patients with IBD. Moreover, by studying the SASP factors associated with enteric gliosis, researchers can gain insights into the mechanisms underlying IBD pathogenesis. This understanding can lead to the identification of new molecular targets for therapeutic intervention. By targeting these mechanisms or specific SASP factors involved in IBD pathogenesis, novel pharmacological interventions can be developed to quell IBD-associated GI inflammation and/or to promote tissue repair. This emphasizes the importance of understanding and targeting the pro-inflammatory effects of glial SASP for more effective therapeutic interventions in IBD management. Notably, serotherapeutic-based interventions hold potential for advancing IBD management by addressing ENS complications and improving patient outcomes. Future studies using senotherapeutic-based interventions are essential to establish the clinical efficacy and applicability of these interventions for IBD patients with enteric gliosis. Therefore, a detailed understanding of the IBD-associated alterations in the ENS could lead to the development of targeted interventions offering novel therapeutic avenues for addressing both GI and mental health aspects of IBD. In summary, studying centric gliosis-associated SASP factors holds promise for the discovery of novel biomarkers and therapeutic targets in IBD. By elucidating the role of these factors in disease pathogenesis and progression, researchers can advance our understanding of IBD and develop more effective strategies for diagnosis, treatment, and patient management.

## Data Availability

No new data were created or analyzed in this study. Data sharing is not applicable to this article.
